# How reliably can algorithms identify eosinophilic asthma phenotypes using non‐invasive biomarkers?

**DOI:** 10.1002/clt2.12182

**Published:** 2022-08-20

**Authors:** Diana Betancor, José María Olaguibel, José Manuel Rodrigo‐Muñoz, Ebymar Arismendi, Pilar Barranco, Blanca Barroso, Irina Bobolea, Blanca Cárdaba, María Jesús Cruz, Elena Curto, Victoria Del Pozo, Francisco‐Javier González‐Barcala, Carlos Martínez‐Rivera, Joaquim Mullol, Xavier Muñoz, Cesar Picado, Vicente Plaza, Santiago Quirce, Manuel Jorge Rial, Lorena Soto, Antonio Valero, Marcela Valverde‐Monge, Joaquin Sastre

**Affiliations:** ^1^ Servicio de Alergología Hospital Universitario Fundación Jiménez Díaz Madrid Spain; ^2^ Servicio de Alergología Hospital Universitario de Navarra Pamplona Navarra Spain; ^3^ CIBER de Enfermedades Respiratorias (CIBERES) Madrid Spain; ^4^ Servicio de Inmunología Instituto de Investigación Sanitaria Hospital Universitario Fundación Jiménez Díaz Madrid Spain; ^5^ Allergy Unit & Severe Asthma Unit, Pneumonology and Allergy Department Hospital Clínic IDIBAPS Universitat de Barcelona Barcelona Spain; ^6^ Servicio de Alergia Hospital Universitario La Paz IdiPAZ Madrid Spain; ^7^ Departamento de Biología Celular, Fisiología e Inmunología Universitat Autónoma de Barcelona Barcelona Spain; ^8^ Departamento de Medicina Respiratoria Hospital de la Santa Creu i Sant Pau Instituto de Investigación Biomédica Sant Pau (IIB Sant Pau) Universidad Autónoma de Barcelona. Departamento de Medicina Barcelona Spain; ^9^ Servicio de Neumología Complejo Hospitalario Universitario de Santiago Santiago de Compostela La Coruña Spain; ^10^ Servicio de Neumología Hospital Germans Trias i Pujol Institut d’Investigació Germans Trias i Pujol Universitat Autònoma de Barcelona Badalona, Barcelona Spain; ^11^ Rhinology Unit & Smell Clinic ENT Department Clinical and Experimental Respiratory Immunoallergy (IDIBAPS) Universitat de Barcelona Barcelona Catalonia Spain; ^12^ Servicio de Neumología Hospital Vall d’Hebron Barcelona Spain; ^13^ Servicio de Alergología Complexo Hospitalario Universitario A Coruña A Coruña Spain

**Keywords:** asthma, biomarkers, eosinophils, exhaled nitric oxide, non‐eosinophilic, phenotypes, sputum

## Abstract

**Background and Aims:**

Asthma is a heterogeneous respiratory disease that encompasses different inflammatory and functional endophenotypes. Many non‐invasive biomarkers has been investigated to its pathobiology. Heany et al proposed a clinical algorithm that classifies severe asthmatic patients into likely‐eosinophilic phenotypes, based on accessible biomarkers: PBE, current treatment, FeNO, presence of nasal polyps (NP) and age of onset.

**Materials and Methods:**

We assessed the concordance between the algorithm proposed by Heany et al. with sputum examination, the gold standard, in 145 asthmatic patients of the MEGA cohort with varying grades of severity.

**Results:**

No correlation was found between both classifications 0.025 (CI = 0.013–0.037). Moreover, no relationship was found between sputum eosinophilia and peripheral blood eosinophilia count in the total studied population.

**Discussion and Conclusion:**

In conclusion, our results suggest that grouping the biomarkers proposed by Heany et al. are insufficient to diagnose eosinophilic phenotypes in asthmatic patients. Sputum analysis remains the gold standard to assess airway inflammation.

## 1 INTRODUCTION

Asthma is a respiratory syndrome characterised by airway inflammation and reversible airway obstruction.^1^ Due to its heterogeneity, considerable efforts have been made to subclassify the disease into different phenotypes and identify non‐invasive biomarkers that reflect its pathobiology. Sputum examination is the gold standard for determining airway inflammation; other non‐invasive biomarkers studied to date, such as peripheral blood eosinophil (PBE) count, serum periostin, fraction of exhaled nitric oxide (FeNO), and serum IgE levels, have low specificity and sensitivity.^2^ Heany et al^3^ proposed a clinical algorithm that classifies severe asthmatic patients based on the likelihood of an eosinophilic phenotype using easily accessible biomarkers such as PBE, current treatment, FeNO, presence of nasal polyps (NP), and age of onset. This algorithm reflects he criteria of the Global Initiative for Asthma (GINA).

We analysed the consistency between the Heany et al. algorithm^3^ and sputum examination in a retrospective analysis of asthmatic patients with varying degrees of severity from eight Spanish hospitals, previously described as the MEGA cohort.^4^ As secondary outcomes, we evaluated the clinical characteristics, asthma severity, and lung function in these phenotypes.^4^, ^5^

## 2 MATERIAL AND METHODS

Patients from the MEGA cohort with a valid sputum analysis and an accurate asthma diagnosis were selected.^4^, ^5^ A total of 145 patients were included. A retrospective observational study was conducted by reviewing the MEGA cohort electronic database. Asthma was diagnosed in patients with an FEV_1_ increase of greater than 200 ml and 12% on spirometry and/or methacholine PC_20_ < 16 mg/ml. Sputum eosinophilia was defined as >3% eosinophils. The ethics committees of each participating hospital approved this study. All subjects provided signed informed consent.

Study data included demographic and clinical characteristics, asthma severity (following GINA guidelines^1^) and control (assessed using the Asthma Control Test), treatment, number of exacerbations, and exacerbation severity. Lung function tests (spirometry and plethysmography), sputum eosinophil count, PBE, and FeNO were also collected at baseline.

Quantitative variables were described as mean and standard deviation, and qualitative variables as absolute and relative frequencies. Inter‐group comparisons were performed using chi‐square test or Fisher exact test for qualitative and ANOVA or Kruskal–Wallis for quantitative variables. Agreement was assessed with the kappa coefficient. Correlations were estimated by Spearman's R. Statistical analysis was carried out using the GraphPad Instat 6 (GraphPad Software). *p* values <0.05 were considered significant.

## 3 RESULTS

Data from 145 asthmatic subjects aged 18–75 years were categorised according to the phenotypes proposed by Heany et al.^3^ Grade 3 (likely eosinophilic) was the most prevalent (69.6%), followed by grade 2 (likely eosinophilic; 20.8%), grade 1 (less likely; 16.8%); and grade 0 (non‐eosinophilic; 5.9%). The average patient age was 48 years, and a majority were female. There was no significant difference in demographic characteristics, asthma severity, exacerbations, or lung function between grades. As FeNO, PBE, and NP were classification criteria, higher levels were shown in grade 3 (*p* < 0.05). Data are summarised in Table 1.

**TABLE 1:** Demographic, clinical, and diagnostic test results of studied patients

	Grade 0 (non‐eosinophilic)	Grade 1 (less likely eosinophilic)	Grade 2 (likely eosinophilic)	Grade 3 (most likely eosinophilic)	
No. subjects (%)	6 (5.9)	17 (16.8)	21 (20.8)	101 (69.6)	
Demographic characteristics					
Individual characteristics					
Female sex, *N* (%)	3 (50)	9 (52.9)	12 (57.1)	66 (65.3)	NS
Mean age, years, mean (SD)	47 (13)	52.1 (11.4)	47 (13)	48 (13)	NS
Body mass index (BMI), mean (SD)	27.2 (5.3)	26.9 (5.3)	26.9 (5.2)	26.8 (5.2)	NS
Obesity,^1^ *N* (%)	2 (33.3)	3 (17.6)	2 (9.5)	16 (15.8)	NS
Residency, urban area, *N* (%)	5 (83.3)	8 (47.1)	16 (76.2)	78 (77.2)	NS (0.06)
Comorbidities, *N* (%)					
Atopy^2^	5 (83.3)	13 (76.5)	19 (90.5)	75 (74.3)	NS
Allergic rhinitis	3 (50)	11 (64.7)	13 (61.9)	58 (57.4)	NS
Bronchiectasis	1 (16.6)	1 (5.9)	2 (9.5)	11 (10.9)	NS
CRSwNP	0 (0)	4 (23.5)	1 (4.8)	52 (51.5)	0.02
CRSsNP	3 (50)	1 (5.9)	4 (19.1)	13 (12.9)	0.05
Obstructive sleep apnoea syndrome (OSAS)	1 (16.6)	0 (0)	1 (4.8)	4 (3.9)	NS
Smoking habit, *N* (%)					
Never smoker	4 (66.6)	9 (52.9)	12 (57.1)	55 (54.5)	NS
Current smoker	0 (0)	2 (17.6)	1 (4.8)	11 (10.9)	NS
Ex‐smoker	2 (33.3)	6 (35.3)	8 (38.1)	31 (30.7)	NS
Education level, *N* (%)					
Higher education	3 (50)	12 (70.6)	11 (52.4)	42 (48.3)	NS
Primary education	1 (16.6)	3 (17.6)	10 (47.6)	44 (50.6)	NS
No studies	2 (33.3)^a^	2 (11.8)	0 (0)^a^	1 (1.1)^a^	<0.0001
Clinical characteristics					
Treatment, *N* (%)					
ICS/LABA	6 (100)	16 (94.11)	19 (90.5)	89 (88.1)	NS
Long‐term OCS	0 (0)	0 (0)	2 (9.5)	14 (13.9)	NS
Asthma severity,^3^ *N* (%)					
Intermittent	0 (0)	0 (0)	1 (4.8)	5 (4.9)	NS
Mild persistent	2 (33.3)	2 (11.8)	4 (19.1)	15 (14.8)	NS
Moderate persistent	2 (33.3)	6 (35.3)	4 (19.1)	28 (27.7)	NS
Severe persistent	2 (33.3)	7 (41.2)	12 (57.1)	53 (52.5)	NS
Exacerbations, *N* (%)					
Patients with asthma exacerbation during previous year	4 (66.6)	6 (35.3)	16 (76.2)	53 (52.5)	NS (0.07)
Severe asthma exacerbation	0 (0)	1 (5.8)	7 (33.3)	16 (15.8)	NS (0.07)
Exacerbations over the previous year, mean (SD)	2.7 (1.2)	1.5 (0.8)	1.9 (1.6)	3.5 (3.7)	NS
Emergency department (ED) visits	1 (1.3)	0.4 (0.8)	0.6 (0.95)	0.6 (1.4)	NS
≥5 ED visits	0 (0)	0 (0)	0 (0)	7 (6.9)	NS
ICU Admission	0 (0)	0.1 (0.3)	0 (0)	0.1 (0.3)	NS
Asthma control in ACT, *N* (%)					
Completely controlled	2 (33.3)	5 (29.4)	7 (33.2)^b^	18 (17.8)^b^	0.01
Well‐controlled	3 (50)	6 (35.3)	9 (42.8)	49 (48.5)	NS
Poorly controlled	1 (16.6)	6 (35.3)	5 (23.8)	34 (33.6)	NS
Respiratory function tests and other biomarkers					
Total IgE, IU/mL, mean (SD)	574.4 (855.2)	417.0 (532.1)	615.0 (171.0)	414.5 (634.9)	NS
Peripheral eosinophilia cells/μL mean (SD)	92.2 (37.9)^c^	103.9 (34.8)^c^	357.1 (347.1)^c^	357.1 (337.3)^c^	<0.001
Spirometry, litres, mean (SD)					
FEV_1_	2.2 (1.1)	2.3 (0.7)	2.7 (0.8)	2.6 (0.8)	NS
FVC	3.1 (1.3)	3.5 (1.1)	3.7 (0.8)	3.6 (0.9)	NS
FEV_1_/FVC	72.4 (10.1)	67.9 (7.6)	71.9 (11.4)	71.2 (9.3)	NS
Positive spirometry bronchodilator test,^4^ *N* (%)	4 (66.7)	4 (23.5)	4 (19.1)	21 (20.8)	
Plethysmography, litres, mean (SD)					
TLC	5.2 (1.5)	6.2 (1.3)	6.8 (0.5)	5.9 (1.1)	NS
RV	2.1 (0.4)	2.3 (0.8)	2.1 (0.7)	2.1 (0.8)	NS
Functional spirometry phenotype,^5^ *N* (%)					
Normal	2 (33.3)	8 (47.1)	10 (47.6)	63 (63.6)	NS
Obstructive	0 (0)	3 (17.6)	5 (23.9)	15 (15.2)	NS
Air trapping	4 (66.7)	6 (35.3)	6 (28.6)	21 (21.2)	NS (0.06)
FeNO, ppb, mean (SD)	18.8 (4.2)^d^	28.8 (22.5)^d^	44.2 (39.7)	55.3 (46.2)^d^	0.03
Methacholine challenge, PC_20_ mean (SD)	0.5 (0.6)	1.8 (2.3)	0.2 (0)	2.0 (2.9)	NS
Sputum analysis					
Sputum eosinophilia, mean (SD)	1.7 (1.8)	8.9 (16.4)	7.6 (15.1)	11.4 (20.2)	NS
Patients with sputum eosinophils >3%, *N* (%)	2 (33.3)	8 (47.1)	11 (52.4)	48 (47.5)	NS
Cellular profile, *N* (%)					
Eosinophilic	1 (16.7)	6 (35.3)	7 (33.3)	45 (44.5)	NS
Mixed	1 (16.6)	2 (11.8)	4 (19.0)^c^	3 (2.9)^c^	0.03
Neutrophilic	1 (16.6)	5 (29.4)	4 (19.0)	10 (9.9)	NS
Paucigranulocytic	3 (50)	4 (23.5)	6 (28.6)	43 (42.6)	NS

Abbreviations: ACT, Asthma Control Test; CRSsNP, chronic rhinosinusitis without nasal polyposis; CRSwNP, chronic rhinosinusitis with nasal polyposis; FEV_1,_ forced expiratory volume during the first second; FVC, forced vital capacity; ICS/LABA, inhaled corticosteroids/long‐acting beta 2‐agonists; ICU, intensive care unit; NS, not statistically significant; OCS, oral corticosteroids; RV, residual volume; TLC, total lung capacity.

^a^ Significant differences were determined between grades 0–2 and 0–3.

^b^ Significant difference between both.

^c^ Significant differences were determined between all inter‐group analysis except 0–1.

^d^ Significant differences were determined between grade 0–3 and 1–3.

^1^ Obesity was defined as BMI over 30 kg/m^2^.

^2^ Atopy was defined as the presence of at least 1 skin prick test or specific serum IgE positive to common allergens.

^3^ Asthma severity was assessed following the Global Initiative for Asthma (GINA) guidelines.

^4^ Positive bronchodilator test was defined as an increase in FEV_1_ of greater than 200 ml and more than 12% of the baseline value 15 min after the administration of 2 puffs of salbutamol.

^5^ Functional phenotype was defined as normal when FEV_1_ >80% and FEV_1_/FVC >70%, obstructive when FEV_1_/FVC <70%, and air trapping when FVC <80% or a 12% FVC increase after bronchodilation.

The agreement between the eosinophilia grades proposed by Heany et al^3^ (grade 2–3) and eosinophilic sputum for the MEGA cohort was 0.025 (95% CI = 0.013–0.037).

**FIGURE 1:**
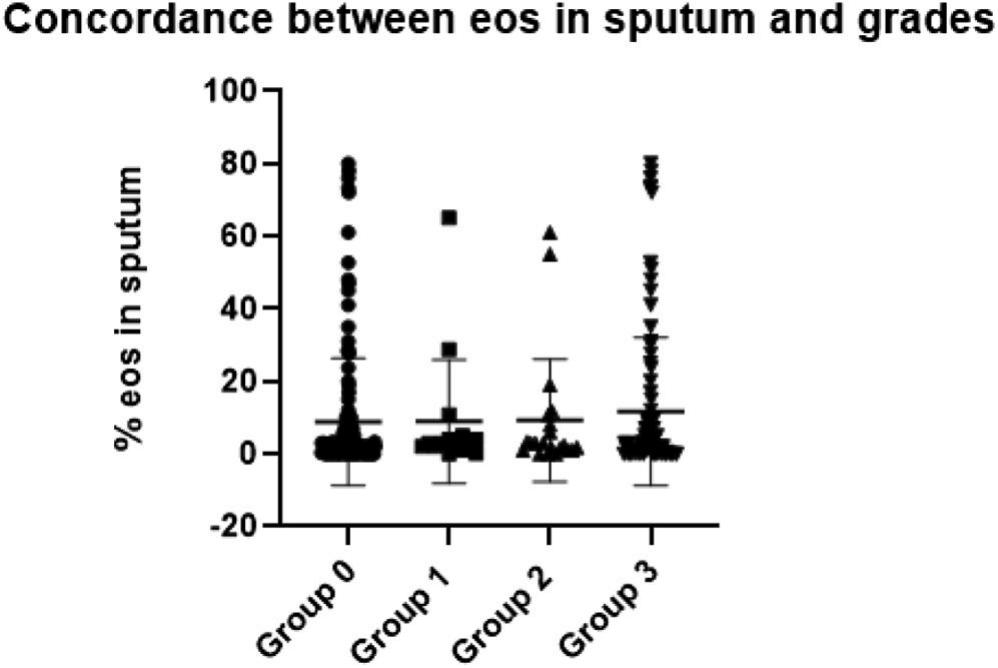
Concordance between values of eosinophils in sputum and Heany et al grades

Though the sputum eosinophilia rate was higher in grade 3, it was not statistically significant compared to the other grades (see Table 1 and Figure 1). We examined the relationship of sputum eosinophils with PBE count, finding no significant correlation in the total population (*r* = 0.11, *p* = 0.22). No correlation was found considering different grades (*r* = −0.17 (*p* = 0.93), *r* = 0.08 (*p* = 0.78), *r* = 0.35 (*p* = 0.12), and *r* = 0.16 (*p* = 0.16)) for grades 0, 1, 2, and 3, respectively.

## 4 DISCUSSION

Several biomarkers have been considered to phenotype asthmatic patients. Heany et al. proposed a system of eosinophilic probability depending on easily accessible biomarkers.^3^ The low agreement found in our study between this algorithm and sputum analysis suggests no relationship between the two criteria.

We observed higher sputum eosinophilia levels in grade 3 of Heany's classification, but without reaching statistical significance. The percentage of patients with sputum eosinophils >3% was similar in grades 2–3 (eosinophilic) and 1, indicating an inability of the proposed system to phenotype these patients.

As in other studies showing a 70%–80% prevalence of the eosinophilic phenotype in tertiary centers,^3^, ^6^ the 69.6% rate in our study contrasts with the 50% previously proposed.^1^ Furthermore, our study found a 5.9% prevalence of non‐T2 asthma, similar to the 5% reported by Kerkhof et al.^7^

Worse lung function is associated with eosinophilic phenotypes,^6^, ^8^, ^9^ as demonstrated in our previous report characterising MEGA patients.^5^ No differences in lung function were found in the present study, likely owing to the presence of sputum eosinophilia in all grades.

A clear correlation has been reported between eosinophilic asthma and greater severity and exacerbations.^6^, ^8^, ^9^ Higher exacerbation rates and severity were found in the “eosinophilic grades” of our study, without reaching a statistical difference (*p* = 0.07).

Some biomarkers used in the Heany classification are easily influenced by external factors, such as treatments and other illnesses in the case of PBE, and age, gender, atopy, and tobacco and food/beverage consumption for FeNO. Wagener et al.^10^ estimated the sensitivity of PBE and FeNO in diagnosing eosinophilic phenotypes at around 89% and 78%, respectively. Nevertheless, Lemière et al.^9^ found no correlation between FeNO and sputum eosinophilia. PBE has been suggested as a sputum eosinophilia predictor,^10^ though we found no correlation between sputum eosinophils and PBE, thus resembling other studies.^2^, ^8^ Due to this external variability, evidence supporting these biomarkers in phenotyping asthma remains unclear.

Kjarsgaard et al demonstrated the presence of free eosinophil granules in the airway in the absence of intact eosinophils.^11^ Detecting intact eosinophils only, as in our study, may give misleading information on the real prevalence of eosinophilic sputum, marking one limitation of our study. Further limitations include a possible bias toward recruiting predominantly eosinophilic patients in tertiary centres and allergy clinics, which could be confounding given the limitations in finding significant differences in unequally sized groups.

In conclusion, our results suggest that the biomarker groupings proposed by Heany et al. are insufficient to diagnose eosinophilic phenotypes in asthmatic patients. Sputum analysis remains the gold standard to assess airway inflammation.

## AUTHOR CONTRIBUTIONS

**Diana Betancor**: Data curation (equal); Formal analysis (equal); Methodology (equal); Writing – review & editing (equal). **Jose Maria Olaguibel**: Data curation (equal); Formal analysis (equal); Investigation (equal); Methodology (equal); Project administration (equal); Writing – original draft (equal); Writing – review & editing (equal). **Jose Manuel Rodrigo Munoz**: Data curation (equal); Resources (equal); Supervision (equal); Validation (equal); Writing – review & editing (equal). **Ebymar Arismendi**: Formal analysis (equal); Resources (equal); Validation (equal); Writing – review & editing (equal). **Pilar Barranco**: Formal analysis (equal); Resources (equal); Validation (equal); Writing – review & editing (equal). **Blanca Barroso**: Data curation (equal); Resources (equal); Writing – original draft (equal); Writing – review & editing (equal). **Irina Bobolea**: Formal analysis (equal); Resources (equal); Validation (equal); Writing – review & editing (equal). **Blanca Cardaba**: Formal analysis (equal); Resources (equal); Validation (equal); Writing – review & editing (equal). **Maria Jesus Cruz**: Formal analysis (equal); Resources (equal); Validation (equal); Writing – review & editing (equal). **Elena Curto**: Formal analysis (equal); Resources (equal); Validation (equal); Writing – review & editing (equal). **Victoria Del Pozo**: Formal analysis (equal); Resources (equal); Validation (equal); Writing – review & editing; (equal). **Francisco‐Javier Gonzalez‐Barcala**: Formal analysis (equal); Resources (equal); Validation (equal); Writing – review & editing (equal). **Carlos Martinez‐Rivera**: Formal analysis (equal); Resources (equal); Validation (equal); Writing – review & editing (equal). **Joaquim Mullol**: Formal analysis (equal); Resources (equal); Validation (equal); Writing – review & editing (equal). **Xavier Munoz**: Formal analysis (equal); Resources (equal); Validation (equal); Writing – review & editing (equal). **Cesar Picado**: Formal analysis (equal); Resources (equal); Validation (equal); Writing – review & editing (equal). **Vicente Plaza**: Formal analysis (equal); Resources (equal); Validation (equal); Writing – review & editing (equal). **Santiago Quirce**: Formal analysis (equal); Resources (equal); Validation (equal); Writing – review & editing (equal). **Manuel Rial Prado**: Formal analysis (equal); Resources (equal); Validation (equal); Writing – review & editing (equal). **Lorena Soto‐Retes**: Formal analysis (equal); Resources (equal); Validation (equal); Writing – review & editing (equal). **Antonio Valero**: Formal analysis (equal); Resources (equal); Validation (equal); Writing – review & editing (equal). **Marcela Valverde**: Formal analysis (equal); Resources (equal); Validation (equal); Writing – review & editing (equal). **Joaquin Sastre**: Data curation (equal); Formal analysis (equal); Methodology (equal); Project administration (equal); Resources (equal); Validation (equal); Writing – original draft (equal); Writing – review & editing (equal).

## CONFLICT OF INTEREST

Dr. Betancor is supported by a Rio Hortega Research Contract from Instituto Carlos III, Spanish Ministry of Science. Dr. Valverde has received lecturing fees from GSK and sits on the advisory board for Organon. Dr. Rial reports receiving personal fees from GSK, Allergy Therapeutics, and AstraZeneca outside the submitted work. Dr. González Barcala reports personal fees from ALK, AstraZeneca, Bial, Boehringer Ingelheim, Chiesi, Gebro Pharma, GlaxoSmithKline, Laboratorios Esteve, Menarini, Mundipharma, Novartis, Rovi, Roxall, Stallergenes‐Greer, Teva, as well as grants from Mundipharma outside the submitted work. Dr. Quirce reports personal fees from AstraZeneca, Novartis, Sanofi, Boehringer Ingelheim, Teva, ALK, Mundipharma, GSK, Chiesi, and Leti outside the submitted work. Dr. Soto reports non‐financial support from CIBER de Enfermedades Respiratorias (CIBERES) during the conduct of the study; outside of the present work, she reports personal fees from Stallergennes‐Greer, Menarini, and Novartis, personal fees from GSK, Hal Allergy, Allergy Therapeutics, AstraZeneca, and grants from Sociedad Española de Alergología e Inmunología Clínica SEAIC and Sociedad Española de Neumología y Cirugía Torácica SEPAR. Dr. Martinez Rivera reports having received grants and personal fees from AstraZeneca, Teva, GSK, Novartis, and Mundipharma outside the submitted work. Dr. Munoz reports personal fees from AstraZeneca, Boehringer Ingelheim, GlaxoSmithKline, Novartis, Teva, Mundifarma, Chiesi, and Faes outside the submitted work. Dr. Sastre reports grants and personal fees from Sanofi, GSK, Novartis, AstraZeneca, Mundipharma, and Faes Farma outside the submitted work. Dr. Olaguibel reports grants from Sanofi and/or personal fees from AstraZeneca and Mundipharma outside the submitted work. Dr. Plaza reports grants and personal fees from AstraZeneca, Boehringer Ingelheim, Merck, Chiesi, Novartis, Menarini, and Sanofi outside the submitted work. Dr. Mullol reports personal and other fees from Sanofi‐Genzyme & Regeneron, Novartis, Viatris (Mylan pharma), Uriach group, Mitsubishi‐Tanabe, Menarini, UCB, AstraZeneca, GSK, and MSD outside the submitted work. Dr. del Pozo reports personal and other fees from Sanofi, AstraZeneca, and GSK outside the submitted work. All other authors have no conflicts of interest.
